# Vegan Alternatives to Processed Cheese and Yogurt Launched in the European Market during 2020: A Nutritional Challenge?

**DOI:** 10.3390/foods10112782

**Published:** 2021-11-12

**Authors:** Fatma Boukid, Melisa Lamri, Basharat Nabi Dar, Marta Garron, Massimo Castellari

**Affiliations:** 1 Food Safety and Functionality Programme, Food Industries, Institute of Agriculture and Food Research and Technology (IRTA), Finca Camps i Armet S/N, 17121 Monells, Spain; massimo.castellari@irta.cat; 2 Laboratory of Food Quality and Food Safety, Department of Food technology, Université Mouloud Mammeri, Tizi-Ouzou 15000, Algeria; lamrimeliza1@gmail.com; 3 Department of Food Technology, IUST, Awantipora 192122, India; darnabi@gmail.com; 4 Food Quality and Technology Programme, Food Industries, Institute of Agriculture and Food Research and Technology (IRTA), Finca Camps i Armet S/N, 17121 Monells, Spain; marta.garron@irta.cat

**Keywords:** vegan, dairy, yogurt, cheese, nutrition, allergens

## Abstract

Vegan alternatives to cheese (VAC) and yogurt (VAY) are fast-growing markets in Europe due to the increasing interest in plant-based alternatives to dairy products. This study aimed to take a closer look at the year 2020 and accordingly retrieved the nutritional information of dairy cheese and yogurt and their vegan counterparts for comparison. It was found that VAY (*n* = 182) provide more energy, total fats, and carbohydrates than dairy yogurt (*n* = 86), while saturated fatty acids (SFAs), sugars, and salt were not different between the two categories. Compared to dairy products (25.6%), 72.9% of the alternative products were declared low/no/reduced allergen, hence providing a larger spectrum of products to respond to consumers’ requirements. VAC (*n* = 114) showed high versatility of form compared to dairy (*n* = 115). Nutritionally, VAC have higher total fats, SFAs, and carbohydrates, but lower protein, salt, and sugar than dairy cheese. Food developers will continue to look for clean label solutions to improve the nutritional values of vegan products through the incorporation of natural ingredients, besides enhancing their taste and texture to appeal to flexitarians.

## 1. Introduction

A plant-based lifestyle is becoming increasingly popular, and people are shifting from an animal-based diet to a plant-based diet [1]. This has boosted investments in the plant-based food sector to become mainstream. One of the main focuses is to create a wide range of alternative products as healthier and more sustainable options than animal-based products [2,3,4]. Although this trend attributes a lot of attention to launching meat analogues, the development of plant-based alternatives to dairy products is becoming popular worldwide [5,6]. Plant-based alternatives to dairy are referred to as drinks, beverages, dairy alternatives, or some other name other than “milk” or “cheese” or “yogurt” [7]. The global market of plant-based alternatives to dairy was estimated to a value of USD 22.6 billion in 2020 and is projected to reach USD 40.6 billion by 2026, recording a compound annual growth rate (CAGR) of 10.3% in terms of value [8]. In Europe, the plant-based alternatives to dairy products market is expected to reach USD 2.22 million by 2026 with a GAGR of 7.12 for the forecast 2021–2026 [9]. This fast growth is driven by increasing prevalence toward allergenicity of cow’s milk, lactose intolerance, changing consumer lifestyles, and interest in alternative diets (i.e., vegan and flexitarian) [10,11]. In 2020, the COVID-19 pandemic accelerated this process since it made consumers rethink their lifestyle and deviate to a more plant-based diet as a healthier option [12]. Therefore, the demand for a variety of vegan alternatives to cheese (VAC) and yogurt (VAY), among others, is gradually gaining importance in the market [8].

VAY represent an important segment among the vegan alternatives. Its global market size was estimated at USD 2.02 billion in 2020 and is projected to grow at a CAGR of 18.9% from 2020 to 2027 [13]. Europe dominates the VAY market with a share of ~50%. On the other hand, the global market of VAC was valued at USD 2.70 million in 2019 [14], and is expected to grow at a CAGR of 8.91% to reach USD 4.58 billion by 2025 [15]. The EU market of these alternatives witnessed huge growth, from 28 million in 2018 to 60 million in 2020.

VAY are generally made by fermentation of aqueous extracts obtained from different raw materials (e.g., legumes, oil seeds, cereals, or pseudo-cereals) using lactic acid bacteria (e.g., *Streptococcus thermophilus* and *Lactobacillus delbrückii* subsp. *bulgaricus*) to obtain a gel structure similar to dairy products [16]. The major challenges faced by producers are associated with the appearance and texture properties caused by the phase separation [2]. Similar to processed dairy cheese, plant-based alternatives are emulsions of oil-in-water, containing protein, added stabilizers, emulsifiers, flavors, colors, preservatives, and water [5]. These ingredients are blended to imitate cheese’s appearance and consistency and mostly no maturation period is needed [17]. Processed cheese may be classified as dairy, partially dairy or hybrid, or non-dairy, depending on whether components are from dairy or plant-based sources [18]. Processed dairy cheese contains a minimum cheese content of 51%, in which non-cheese ingredients (e.g., dairy ingredients) may be used at levels up to ~15% [19]. Hybrid products contain both non-dairy and dairy ingredients to impart a cheese flavor. However, vegan products contain no added cheese or any dairy ingredients, and consequently, producing a smooth and uniform texture similar to that of dairy is challenging [17,20].

The denomination of vegan alternatives to dairy products similarly to meat alternatives is a controversial subject among the different players in the food industry. This debate raised questions about the nutritional composition of alternatives compared to conventional products and therefore the use of words like “milk”, “cheese”, and yogurt” for the labeling of vegan alternatives might mislead consumers. Regulation (EU) No. 1169/2011), article 36 (3) (b) mentions information “related to the suitability of a food for vegetarians or vegans” within the list of voluntary food information. Nevertheless, it does not provide a clear definition or/ and requirements for “vegan” or vegetarian” for a food product. The European food and drink industry and the European Vegetarian Union submitted a joint position titled “Mandatory food labelling Non-Vegetarian/Vegetarian/Vegan” [21]. In 2017, the European Court of Justice decided to prohibit the use of dairy-like terms in labeling alternative products [22].

Beside product denomination, nutritional labeling can provide crucial information like nutritional composition, ingredients, and allergens that can help consumers in making an informed choice at the point of sale [23]. In the EU, Regulation (EU) 1169/2011 dedicated to food labeling requires declaration of the energy, total and saturated fats, carbohydrates, sugars, proteins, and salt in prepacked foods [23]. Ideally, plant-based alternatives should possess a similar nutritional profile to dairy products, while also resembling them in taste, texture, and appearance [24]. From a nutritional point of view, it should be emphasized that the nutritional value of plant-based beverages differs from that of cow’s milk [25,26,27]. Compared to cow’s milk, plant-based beverages have lower protein, calcium, and vitamin D but higher added salt. It is well known that plant proteins have lower amounts (or lack) of essential amino acids and are less digestible and bioavailable compared to animal counterparts [28]. However, amino acid complementation (mixing of different plant proteins), with a frequent consumption of plant-based products [28], and proper product development and processing of the plant-based constituents [29] are being used as alternative strategies to maximize the essential amino acid contents of plant-based foods [30]. Vegan alternatives to milk provide more energy than milk attributable to their high total fats and carbohydrates. Besides the energy source, healthy foods should satisfy the need for essential nutrients [31]. Therefore, reformulating vegan alternatives to dairy products requires taking into account the compensation of calcium, magnesium, and vitamin D deficits through adequate fortifications [10,32].

Nevertheless, in the case of VAY and VAC, less information is currently available about their nutritional composition and how the absence of dairy ingredients can be overcome from sensorial and technological perspectives. Another challenge for alternative products to dairy is the allergenic potential of the plant-based ingredients, such as soy, wheat, pea, faba, peanut, lupine, and chickpea [33]. For genetically predisposed subjects, the intake of such ingredients can provoke symptoms ranging from mild to severe (enterocolitis atopic eczema and immediate IgE-mediated reactions) [11]. Furthermore, plant sources have been reported to have cross-reactivity. For instance, peanut allergy can be associated with an allergy to lentil, chickpea, and pea [34]. This is an important issue to be carefully considered when formulating alternatives products, such as milk, yogurt, and cheese.

Although different studies have focused on comparing cow milk to plant-based alternatives, VAY and VAC have been scarcely investigated. Furthermore, to the authors’ best knowledge, no research has addressed both the nutritional composition and allergenicity of these dairy alternatives in the same study. Nevertheless, these aspects are crucial for consumers to make their choice about purchasing food products. This implies that from a food developer perspective, both nutritional expectations and potential allergens are determining factors when designing the product and deciding the list of ingredients. Thus, the aim of this work was to answer the question of whether these alternatives can be considered more nutritionally equilibrated than traditional products. To be exhaustive, this study did not focus on a benchmark of commercial products but included all product launches of the year 2020 in the EU market. Indeed, the alternative products market is dynamic, and focusing the interest on the newest products launched in the market will offer an updated image of the current direction of new food development. All products with complete mandatory nutritional information on their labels in concordance with EU Regulation 1169/2011 [23] were retrieved and compared to dairy products launched during the year 2020.

## 2. Materials and Methods

Data collection: The search for alternatives products to yogurt and cheese was carried out, on 21 May 2021, by consulting the Mintel Global New Product Database (Mintel GNPD-Mintel Group Ltd., London, UK, https://portal.mintel.com/portal/ accessed on 21 May 2021), with a focus on 2020 launches in Europe (44 countries including UK). Out of the super-category of “foods”, the search was concentrated on the category “dairy”. The Mintel GNPD search was conducted using the search parameters specified in Table 1. For VAY, products were included with the subcategory “plant-based spoonable yogurts (dairy alternatives)”, with a total of 229 products. To ensure that only vegan products were retrieved, and no hybrid products (made from plant-based and animal-based ingredients) were on the list, the claim “vegan/ non animal ingredients” was added to the list of filters. This resulted in a list of 209 products, and after adding the mandatory list of nutrients based on the EU regulation as a filter, a list of 182 products was obtained. Natural yogurt products were included within the subcategory of “spoonable yogurt” (with a total of 1478 products). When adding “natural” as the name of the product, we obtained a list of 114 products. By adding the mandatory nutritional facts as another criteria, we obtained a list of 86 products. For VAC, products were included within the sub-category processed cheese (containing 509 products). To ensure only vegan products were retrieved, the claim “vegan/ non animal ingredients” was added to the list of filters, resulting in 123 products, and when all mandatory nutritional information was added as a filter, only 114 products were retrieved. As for dairy cheese, we added “milk” as an ingredient as a filter, resulting in 155 products, and when all mandatory nutritional information was added as a filter, only 115 products were obtained. The results of all searches were exported to Microsoft Excel (Microsoft Office, Washington, WA, USA) (Table S1).

Data extraction: Following each search, the mandatory nutritional labeling of all products: energy (kcal/100 g), total fat (g/100 g), saturated fatty acids (SFAs) (g/100 g), carbohydrates (g/100 g), sugars (g/100 g), protein (g/100 g), and salt (g/100 g), was retrieved. Furthermore, a list of ingredients, allergies, and claims was retrieved.

Statistical data analysis: The statistical analysis was carried out using the Statistical Package for Social Sciences software (IBM SPSS Statistics, Version 25.0, IBM Corp., Chicago, IL, USA). Based on the Kolmogorov–Smirnov test, the normality of the data distribution was rejected, and therefore data were expressed as median values with interquartile ranges. Energy and nutrient contents per 100 g of products were analyzed using Kruskal–Wallis non-parametric one-way ANOVA for independent samples with multiple pairwise comparisons and the Mann–Whitney non-parametric test for two independent samples.

## 3. Results and Discussion

### 3.1. Nutritional Composition

#### 3.1.1. Vegan Alternatives to Yogurt

In 2020, 182 new VAY products were launched in the EU market, showing the rising trend of consuming plant-based products that has boosted food companies to enlarge their portfolios in response to consumer demand. The total launches of dairy yogurt reached 1225 items including 86 natural dairy yogurts. These items were retrieved for comparison with VAY.

**Figure 1:**
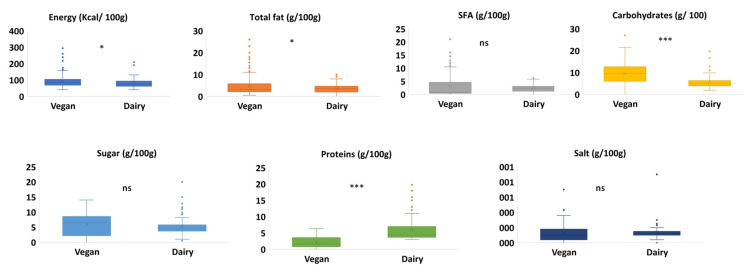
Nutritional profile of vegan alternatives to yogurt (n = 182) vs. natural dairy yogurt (n = 86). The box-plot legend: the box is limited by the lower (Q1 = 25th) and upper (Q3 = 75th) quartile; the median is the horizontal line dividing the box; whiskers above and below the box indicate the 10th and 90th percentiles; outliers are the points outside the quartile 10–90th percentiles. *: *p* < 0.05, ***: *p* < 0.001, ns: non-significant (*p* > 0.05).

Figure 1 illustrates the results of the mandatory information retrieved from the “nutritional information” section on the front package of VAY and natural yogurt. A significant difference in fat, carbohydrates, and energy content was observed. The high-fat content in VAY is attributed to the high amount of fats used, such as coconut, cashew, and almond, to attain the desired texture and consistency [35]. However, the flavoring role of animal fat is substituted by added flavoring substances (trisodium citrate, citric acids, lemon juice concentrate, malic acid, lactic acids) (Table 2). Among these ingredients, almond (*n* = 22) and coconut (*n* = 82) are the most used, while vegetable oils (*n* = 33) including sunflower seed oil and rapeseed oil are used to a lesser extent. In recent years, the consumption of fat products has decreased due to the awareness of the probable harmful effect of fat on consumers’ health; thus, the dietary habits of consumers have changed and market interest has moved in favor of low- or non-fat dairy products [36]. Subsequently, there is a tendency to reduce the fat amount in vegan and dairy categories, where 22.1% (*n* = 40) of non-dairy and 23.3% (*n* = 20) of dairy yogurts claimed to be low/no/reduced fat. High carbohydrates in non-dairy are derived from the use of sugars and starches (native and modified). Starches have an important role in obtaining an appropriate viscosity, sensory properties, and inhibiting/reducing wheying-off during storage and transportation, as well as boosting the ratio of total solids of non-dairy products [2,36].

Saturated fatty acids (SFAs) and sugars did not show significant differences between both groups. Notably, VAY had a higher intra-variability of SFA due to the diversity of the fats used (types and composition) in vegan products (Table 2). For instance, fats derived from coconut contain around 82.5 g/100 g SFAs compared to palm oil (49.3), olive oil (13.8), or nuts (3.8) [37]. Dairy products showed more homogeneity because of the low SFAs in cow’s milk (1.9 g/100 g) [37]. This might also be in part attributed to the difference in the number of VAY products (*n* = 182) and natural dairy yogurt products (*n* = 86). Salt did not vary significantly between dairy and non-dairy products. However, and as expected, proteins were significantly higher in dairy yogurt, where 16.3% (*n* = 14) of the total products claimed to have high/added protein. In non-dairy alternatives, soy is the most used source of protein (*n* = 55), followed by oat (*n* = 34) and pea (*n* = 11). Plant proteins have different compositions and structures than casein and consequently behave differently under acid conditions, resulting in the formation of a non-continuous weak gel [2]. For this reason, hydrocolloids are incorporated to improve the structure formation and to imitate as much as possible the characteristics of a dairy-based yogurt. Commonly, combinations of ingredients (e.g., natural gums, proteins, starches, pectin, and agar) are employed to provide acceptable/desirable textures. Nevertheless, many consumers reject these additives in products because of the growing clean label trend [38]. One possible method to avoid additives is the use of bacteria, mainly lactic acid bacteria and probiotics (Table 2), that produce exopolysaccharides, which can increase disulphide bonding, resulting in the formation of a dense network similar to that of dairy yoghurt [24,39].

#### 3.1.2. Vegan Alternatives to Processed Cheese

In 2020, all launched VAC were found within the processed cheese category and none were included in the hard or soft cheese categories. Therefore, only dairy cheese from the category of processed cheese was retrieved to be compared to vegan products. The results showed that launches of VAC reached 114 items, while dairy launches had 115 new items. These products come in different forms to satisfy different uses and applications. The most common forms are blocks, slices, and spreads in both categories. Noteworthy, VAC products are versatile, showing more diversity in their form, such as shredded, whole, wedges, and balls. This reflects the focus on convenience in research and development to offer versatile vegan products [20,40].

**Figure 2:**
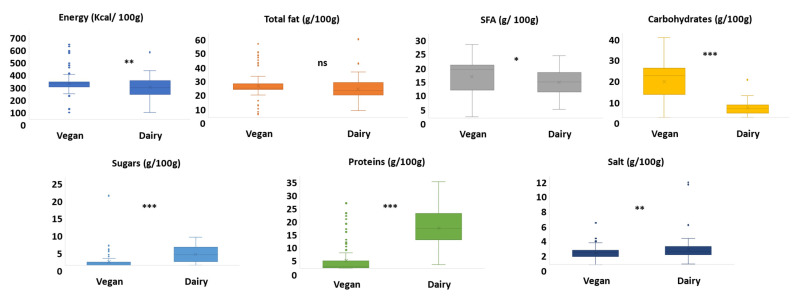
Nutritional profile of vegan alternatives to processed cheese (*n* = 114) vs. dairy processed cheese (*n* = 115). The box-plot legend: the box is limited by the lower (Q1 = 25th) and upper (Q3 = 75th) quartile; the median is the horizontal line dividing the box; whiskers above and below the box indicate the 10th and 90th percentiles; outliers are the points outside the quartile 10–90th percentiles. *: *p* < 0.05, **: *p* < 0.01, ***: *p* < 0.001, ns: non-significant (*p* > 0.05).

Figure 2 illustrates the results of the mandatory information retrieved from the “nutritional information” section on the front package of VAC and processed cheese. VAC provide higher energy than dairy cheese. Although both vegan and dairy products are made with different types of fats (Table 3), total fat content did not vary significantly. Two alternative products claimed low/no/reduced fat, while none claimed to be from the dairy category. Coconut oil is a key ingredient in VAC, while dairy products are mostly made with animal fats or with blends of animal fats and vegetal oils. Almond paste and peanut oil are emerging ingredients as better alternatives to coconut oil since they have lower SFAs and produce a comparable texture and taste [41]. SFAs were found to be significantly higher in alternatives products due to the use of SFA-rich vegetable oils, such as palm oil. Nevertheless, around 10.5% (*n* = 12) of VAC products claimed to be palm oil free. To achieve the desired firmness in a cheese matrix, hydrogenated vegetable oils are used [42]. However, the hydrogenation of vegetable oil increases the content of SFAs and trans-isomeric forms and increases the risk of cardiovascular and heart diseases [43,44,45,46].

Carbohydrates were higher in non-dairy alternatives since starches in their native and modified forms are one of the most used ingredients in vegan cheese formulations [20,47]. However, the sugar content was higher in processed dairy cheese. Indeed, both products are made with high amounts of starches and emulsifiers to obtain the desired texture and taste. The protein content of VAC was significantly lower than in dairy products. Noteworthy, two VAC products claimed a high protein content, showing the direction of product innovation toward diversification of the source of proteins (Table 3) and an increase of their content. The salt content was lower in VAC compared to processed cheese due to the relevant role that salt plays in the final texture product. Besides, the application of enzymes, acids, or heat treatments can further improve the textural acceptability and spreadability of dairy cheese and vegan alternatives [5,48,49].

In response to the market trend of clean label ingredients, 19.3% (*n* = 22) of vegan products claimed to have no additives/preservatives, 23.7% (*n* = 27) were organic, and 19.3% (*n* = 22) GMO free. The nutritional improvement of vegan alternatives was reached through the addition of vitamins and minerals, where 26.3% (*n* = 30) of products claimed to be vitamin/mineral fortified, and 10.5% (*n* = 12) had added calcium.

### 3.2. Allergens

#### 3.2.1. Vegan Alternatives to Yogurt

Besides the “vegan” suitability claim (100.0%; *n* = 181), product labels show other different related claims including “plant based” (72.9%; *n* = 132)), “vegetarian” (54.7%; *n* = 99)), “low/no/reduced lactose” (52.5%; *n* = 95), and “dairy-free” (26.0%; *n* = 47). In front of unclear legislation, food manufacturers use several claims to underline that the product is suitable for vegans. Table 4 illustrates the list of allergens declared on each category in concordance with Regulation (EU) No. 1169/2011. Overall, 72.9% (*n* = 132) of alternatives were declared as “low/no/reduced allergen”, while only 25.6% (*n* = 22) of the yogurts had the same claim. This underlines the current direction in new product development aiming to offer a wide range of alternative products responding to consumers’ needs [50]. Soybean was found to be the most frequent allergen in the non-dairy yogurt-like category [37.60% (*n* = 68)], while no soybean-derived ingredients were used in dairy products. Milk is the main allergen found in dairy products. Indeed, milk allergy is one of the most common food allergies, requiring the complete elimination of milk and derived products from the diet [51]. Nuts were declared in both categories but more broadly in VAY. Gluten and wheat were also declared in vegan and dairy products. On the other hand, 49.7% of the total VAY were declared to be gluten-free (*n* = 90), while dairy gluten-free products were estimated to be 14.0% (*n* = 12)

#### 3.2.2. Vegan Alternatives to Processed Cheese

Besides the claim “vegan”, cheese-like products had other related claims including 51.8% (*n* = 59) “dairy-free”, 46.5% (*n* = 53) “lactose-free”, 34.2% (*n* = 39) “plant-based”, and 33.3% (*n* = 38) “vegetarian”. The list of allergens is summarized in terms of percentages for each type of product in Table 4. Overall, 57% (*n* = 65) of VAC claimed to be allergen-free compared to dairy products with 17.4% (*n* = 20). This confirms the effort made to formulate an alternative product to be suitable to a wider consumer by avoiding allergens. Nuts were the highest allergens declared in non-dairy alternatives since almonds, hazelnuts, walnuts, cashews, pecan nuts, and pistachio nuts are widely used in product formulation [47,52]. However, no product within the dairy category made this claim.

Gluten and wheat were declared in dairy and vegan categories as they act as flavoring and thickening agents [4,53]. Noteworthy, 67.5% (*n* = 77) of the non-dairy products were declared to be gluten-free in response to the rising prevalence of gluten-related disease, while none made this claim in the dairy category. Likewise, soybean was found in both categories, as it is a common ingredient used as a source of proteins. Finally, mustard was declared in both categories since it is used as a flavoring agent. Dairy cheese had a high number of animal allergens including eggs, contributing to the modulation of the consistency of processed cheese [54]. Celery was used as a flavoring agent, while sesame oil contributes to obtaining the desired texture of dairy products [55]. Sulphur dioxide/sulphites were also exclusively declared in dairy cheese since they are used as additives (E 220–228) for product preservation [56].

## 4. Conclusions

Based on the nutritional information retrieved from the labeling of products launched in 2020, VAY and VAC differed nutritionally from their dairy counterparts. VAY provide more energy, total fats, and carbohydrates than dairy yogurt but show similar SFAs, sugars, and salt. On the other hand, VAC have higher fat, SFAs, carbohydrates but lower protein, salt, and sugar contents compared to dairy cheese. The main limitation of alternative products is their low protein content and thus may not represent a healthy choice compared to dairy-based products. Furthermore, retrieval of the list of allergens on products identified that VAY have soybeans, nuts, gluten, and wheat as their main allergens, while VAC have nuts, wheat, gluten, soybeans, and mustard as their main allergens. The analysis of the results of this study allowed the recognition of the quality characteristics of plant-based yogurts and cheese and emphasized important relationships between such characteristics and their formulations that can be utilized in future product development. Vegan alternatives remain a key research area for nutritionists and food technologists for new product development to be used as substitutes for people who are sensitive to the various components of dairy-based products or have some religious bindings, and to meet the consumer’s sensory expectations. Delivering comparable functionality, nutrition, and taste to dairy products will drive the expansion of the dairy alternative market. One of the key challenges is the need for clean labels and hypoallergenic ingredients to substitute the additives commonly used to improve the structure and taste of alternative products.

## Figures and Tables

**Table 1:** Search strategy used on the Mintel Global New Product Database.

Criteria	1st Search	2nd Search	3rd Search	4th Search
Sub-Category	Plant-based spoonable yogurts (dairy alternatives)	Spoonable yogurt	Processed cheese	Processed cheese
Filter	-	Name of the product: Natural	-	Ingredient search: milk
Claim	Vegan/no animal ingredients	-	Vegan/no animal ingredients	
Region	Europe			
Date	Last complete year			
Nutrition	Carbohydrates (listed on pack); Sugars (listed on pack); Protein (listed on pack); Fat (listed on pack); Salt (listed on pack); Energy (kcal) (listed on pack); Saturated Fat (listed on pack)			

“-“: no filter was applied.

**Table 2:** List of ingredients used to make dairy yogurt and their vegan alternatives.

Ingredients	Vegan Products	Dairy Products
Proteins	Soybean, oat, pea protein,	Milk proteins
Fat	Coconut milk/ fat/ paste, almond paste, sunflower seed oil, rapeseed oil	Milk, skim milk, yogurt cream, skimmed milk powder, full fat milk
Carbohydrates	-Sugars: white sugar, unrefined natural sugar glucose, fructose syrup -Starches (native and modified): cassava; tapioca, corn, potato -Dextrins	-White Sugar -Modified Starches
Flavor	-Vanilla, fruit and fruit products (strawberry, blueberry, raspberry, mango) -flavoring substances (trisodium citrate, citric acids, lemon juice concentrate, malic acid, lactic acids)	
Bacterial Cultures	*Streptococcus thermophilus*, *Lactobacillus delbrueckii* ssp. *bulgaricus*, *Bifidobacterium,* *Lactococcus lactis* ssp. lactis, *Lactobacillus acidophilus*	*Bifidobacterium animalis*, *Streptococcus thermophilus*, lactic acid bacteria
Food Thickeners	Pectins, guar gum, inulin, agar, carob bean gum	
	Vitamins (B2, B12, C, D, and E), antioxidants, calcium (tricalcium phosphate, calcium citrates)	Vitamin D

**Table 3:** Main ingredients of processed cheese.

Ingredients	Vegan Products	Dairy Products
Proteins	Pea protein, soy protein, lentil protein	Whey protein, milk protein hydrolysate, casein
Fat	Coconut oil, almond paste sunflower oil, olive oil	Milk, skim milk, butter milk, high condensed fat milk, sweet cream, solid yogurt, cheese, palm oil
Carbohydrates	-modified (potato, corn, cassava) -native (corn starch, cassava, potato, tapioca) -corn flour, rice flour	modified starch, corn starch, what flour, modified potato starch
Flavors	Yeast extracts, paprika, parsley, garlic powder, spirit vinegar, basil leaves	paprika extract, bakery yeast
Emulsifiers	Carrageenan, oat fiber, carob bean gum, xanthan gum, cellulose	sodium polyphosphates, sodium citrates, inulin (chicory root fiber), emulsifying salts (triphosphates, polyphosphates)
Vitamins	B12, vitamin C	

**Table 4:** List of allergen sources declared on yogurt and cheese and their vegan alternatives (VAY and VAC) launched in the EU market in 2020.

VAY	Vegan Products	Dairy Products
Soybeans	37.60% (*n* = 68)	-
Milk	-	90.70% (*n* = 78)
Nuts/Tree Nuts	16.60% (*n* = 30)	2.30% (*n* = 2)
Cereal containing gluten	14.90% (*n* = 27)	2.30% (*n* = 2)
Wheat	0.55% (*n* = 1)	1.20% (*n* = 1)
**VAC**	**Vegan Products**	**Dairy Products**
Nuts/Tree Nuts	19.30% (*n* = 22)	-
Milk	-	94.80% (*n* = 109)
Eggs	-	12.20% (*n* = 14)
Wheat	3.50% (*n* = 4)	10.40% (*n* = 12)
Cereal containing Gluten	13.20% (*n* = 15)	10.40% (*n* = 12)
Soybeans	6.10% (*n* = 7)	1.70% (*n* = 2)
Sulphur Dioxide/Sulphites	-	8.70% (*n* = 10)
Mustard	0.88% (*n* = 1)	6.10% (*n* = 7)
Sesame Seeds	-	3.50% (*n* = 4)
Celery	-	0.87% (*n* = 1)

“-“: no products were retrieved.

## Data Availability

Not applicable.

## Supplementary Materials

The following are available online at https://www.mdpi.com/article/10.3390/foods10112782/s1, Table S1: Nutritional profile of vegan alternatives to cheese and yogurt and dairy products launched in 2020 and commercialized in the EU market.
